# Editorial: Neuroblastoma: Signal Crosstalk and Future Insight Into Clinical Trials

**DOI:** 10.3389/fonc.2022.861963

**Published:** 2022-03-04

**Authors:** Kiyohiro Ando, Takaomi Sanda, Yusuke Suenaga, Tao Liu

**Affiliations:** ^1^Research Institute for Clinical Oncology, Saitama Cancer Center, Saitama, Japan; ^2^Cancer Science Institute of Singapore, National University of Singapore, Singapore, Singapore; ^3^Department of Molecular Carcinogenesis, Chiba Cancer Center Research Institute, Chiba, Japan; ^4^School of Women’s and Children’s Health, University of New South Wales Sydney, Sydney, NSW, Australia; ^5^Children’s Cancer Institute, Sydney, NSW, Australia

**Keywords:** neuroblastoma, epigenetic regulation, noncoding RNA (ncRNA), copy number variation (CNV), intracranial metastasis (IM)

Unfavorable neuroblastoma subtypes with poor survival, because of treatment resistance and metastatic spread, underscore the need for alternative diagnostic and therapeutic approaches. Novel strategies against neuroblastoma heterogeneity are based on investigations of genetic/epigenetic aberrations, mechanistic insights into signal crosstalk, and alterations of immune-related genes for future proof-of-concept clinical trials.

## Novel Therapeutic Proposals Based on the Signal Transduction Cascade and Crosstalk Between Neuroblastoma Oncogenes

Biegel et al. proposed the strategy for suppression of neuroblastoma cell proliferation by inhibiting IGF2BP1, a candidate oncogene located in the frequently gained 17q21 region. Either the single agent BTYNB, an inhibitor of IGF2BP1–RNA binding, or a combination with a certain type of chemotherapeutic agent that suppresses DNA synthesis and CDK inhibitors might synergistically affect the transcriptional regulation of cancer-related pathways (e.g., TP53, PI3K-AKT, JUN, ERK1/2, and STAT3) in a manner similar to that of standard chemotherapeutics.

## Novel Combination Treatment Strategies With Currently Approved and/or Developing Drugs, Particularly Conceptualized Around Metabolic and Epigenetic Aberrations in a Tumor-Agnostic Manner

In a continuation of their previous study, Hu et al. showed that the combination treatment with HMGCR inhibitors (statins) and CYP17A1 (abiraterone acetate) significantly inhibited the tumor growth of SH-SY5Y xenografts by inhibiting the AR-SCAP-SREBPs-CYP17/HMGCR axis. Therefore, blockade of cholesterol and androgen synthesis has emerged as a potential approach for treating neuroblastomas, with reduced adverse effects.

Hanna et al. extended their previous report showing that targeting HSP90 might provide therapeutic benefits for patients with neuroblastoma. They proposed that 17-AAG, an inhibitor of HSP90, could abolish tumor-promoting function mechanistically through multi-protein crosstalk. Furthermore, EI-Soussi et al. described the inhibitory role of ONC201 and ONC206, small-molecule imipridones, in neuroblastoma-related tumorigenic proteins. Although the molecular targets of 17-AAG and ONC201/ONC206 are distinctly different, it is noteworthy that these compounds may affect chromatin remodeling and stem cell self-renewal through the regulation of HMGA1 and L1CAM, respectively, implicating further potential for targeting epigenetic regulation and reprogramming of neuroblastoma.

## Predictive and Therapeutic Value of Non-Coding RNAs in Neuroblastoma

A review by Rezaei et al. described that a series of dysregulated ncRNAs, including miRNAs, lncRNAs, and circRNAs, concomitant with frequent chromosomal ablations, as well as SNPs within ncRNAs, were associated with neuroblastoma pathogenesis. Although efficient drug delivery of these transcripts remains to be resolved, targeting ncRNAs may not only offer a potential for prognosis, but also open a novel therapeutic avenue for neuroblastoma patients.

Zhong et al. proposed the prognostic value of immune-related signatures based on the expression of protein-coding genes and lncRNAs in neuroblastoma. These characteristics suggest that neuroblastoma patients with poor prognosis might be in an immunosuppressed state and may offer promise for improving immunotherapy for neuroblastoma.

## Novel Strategies to Screen Drugs for Molecular Targeting in Neuroblastoma Experimental Models

Wong et al. attempted to integrate profiling of copy number variations (CNVs) in neuroblastoma with drug screening of corresponding patient-derived cell culture models to exploit personalized therapy. They found that the copy number gains of the PI3K and STAT family genes on chromosome 17q were highly susceptible to the targeted inhibitors of PI3K and the cell cycle, suggesting a possible strategy for CNV-based quantitative treatment response prediction.

## Early Detection of Neuroblastoma With Intracranial Metastases

Liu et al. retrospectively investigated the clinical characteristics of 22 neuroblastoma patients with IM, which rarely occur but present serious complications. These patients tended to be incidentally diagnosed by routine physical examination at enterocoelia as the primary site, suggesting the importance of central nervous system (CNS) screening in neuroblastoma patients.

In conclusion, the emerging evidence implicates not only novel therapeutic strategies but also prognostic values, which target candidate oncogenic alterations in neuroblastoma, including IGF2BP1-RNA binding, cholesterol and androgen synthesis, chromatin remodeling, stem cell self-renewal, and dysregulated ncRNAs. Further insightful observations regarding the pathogenesis of individual tumors might highlight small subsets of patients with unfavorable neuroblastomas who may benefit from novel strategies based on currently approved and/or developing drugs.

## Author Contributions

All authors listed have made a substantial, direct, and intellectual contribution to the work and approved it for publication.

## Conflict of Interest

The authors declare that the research was conducted in the absence of any commercial or financial relationships that could be construed as a potential conflict of interest.

## Publisher’s Note

All claims expressed in this article are solely those of the authors and do not necessarily represent those of their affiliated organizations, or those of the publisher, the editors and the reviewers. Any product that may be evaluated in this article, or claim that may be made by its manufacturer, is not guaranteed or endorsed by the publisher.

